# Sites of blood collection and topical antiseptics associated with contaminated cultures: prospective observational study

**DOI:** 10.1038/s41598-021-85614-7

**Published:** 2021-03-18

**Authors:** Koshi Ota, Koji Oba, Keisuke Fukui, Yuri Ito, Emi Hamada, Naomi Mori, Masahiro Oka, Kanna Ota, Yuriko Shibata, Akira Takasu

**Affiliations:** 1 Department of Emergency Medicine, Osaka Medical College, 2-7 Daigaku-machi, Takatsuki City, Osaka, 596-8686 Japan; 2 Department of Biostatistics, School of Public Health, Graduate School of Medicine, The University of Tokyo, Tokyo, Japan; 3 Research and Development Center, Osaka Medical College, Osaka, Japan; 4 Department of Nursing, Osaka Medical College Hospital, Osaka, Japan; 5 Department of Clinical Laboratory, Osaka Medical College Hospital, Osaka, Japan

**Keywords:** Microbiology, Bacteria, Infectious-disease diagnostics, Bacterial infection

## Abstract

We aimed to determine whether puncture sites for blood sampling and topical disinfectants are associated with rates of contaminated blood cultures in the emergency department (ED) of a single institution. This single-center, prospective observational study of 249 consecutive patients aged ≥ 20 years proceeded in the ED of a university hospital in Japan during 6 months. Pairs of blood samples were collected for aerobic and anaerobic culture from all patients in the ED. Physicians selected puncture sites and topical disinfectants according to their personal preference. We found 50 (20.1%) patients with potentially contaminated blood cultures. Fifty-six (22.5%) patients were true bacteremia and 143 (57.4%) patients were true negatives. Multivariate analysis associated more frequent contamination when puncture sites were disinfected with povidone-iodine than with alcohol/chlorhexidine (adjusted risk difference, 12.9%; 95% confidence interval [CI] 8.8–16.9; *P* < 0.001). Sites of blood collection were also associated with contamination. Femoral and central venous with other sites were associated with contamination more frequently than venous sites (adjusted risk difference), 13.1% (95% CI 8.2–17.9; *P* < 0.001]) vs. 17.3% (95% CI 3.6–31.0; *P* = 0.013). Rates of contaminated blood cultures were significantly higher when blood was collected from femoral sites and when povidone-iodine was the topical antiseptic.

## Introduction

Blood cultures are indispensable to detect life-threatening bacteremia, which is associated with high morbidity and mortality rates. Accurate findings of cultured blood samples play important roles in the diagnosis of potentially fatal infections. Contaminated blood cultures that are actually false-positive can result in unnecessary antibiotic use, increased health care costs and most importantly, lead to antimicrobial resistance^1,2^. Several strategies have been recommended to reduce rates of blood culture contamination^3–5^. However, a meta-analysis has determined that only sampling from separate venipuncture sites and a well-trained phlebotomy team can achieve this^6^. Although topical 1.0% alcohol/chlorhexidine gluconate (ACHX) reduces blood culture contamination more effectively than 10% aqueous povidone-iodine (PVI)^7,8^, both agents are routinely applied at our institution as topical disinfectants before blood sampling. The rates of false-positive cultures are significantly reduced when blood is sampled from various venipuncture sites compared with intravenous or central venous catheters^9,10^. Nonetheless, physicians at our institution may sample blood from various sites, such as intravenous and central venous catheters, as well as femoral arteries and veins according to personal preference. Those in our emergency department (ED) tend to sample blood from femoral arteries or veins, yet little is understood about associations between puncture sites for blood sampling and false-positive blood cultures.

Thus, we aimed to determine whether puncture sites for blood sampling and topical antiseptics are associated with blood culture contamination in a single ED.

## Methods

### Study design

This single center, prospective observational study proceeded at the ED of a university hospital in Japan between August 1, 2018 and January 31, 2019. The hospital is an 882-bed university teaching hospital with 8,000 adults presenting at the ED annually. Strengthening the Reporting of Observational studies in Epidemiology (STROBE) guidelines were used to design and report the results of this study. The Institutional Review Board at Osaka Medical College approved the study protocol (675(2476)) and waived the need for written, informed consent.

### Patients

This study included 249 consecutive patients aged ≥ 20 years from whom blood was sampled in the ED. The exclusion criteria comprised blood sampled elsewhere and age < 20 years. If one pair of blood samples was collected at our ED and another was collected elsewhere or not collected, then only the pair collected at our ED was analyzed. One or more of the following comorbidities of the patients were recorded: malignancy, diabetes mellitus, hypertension, prior stroke, dementia, chronic renal insufficiency, liver cirrhosis and coronary artery disease^11–14^.

### Blood cultures

Nurses and other medical staff at our institution are not permitted to collect blood culture samples. Only physicians, especially first- or second-year interns, are permitted to collect blood samples in the ED.

Blood (14–20 mL) from peripheral veins or arteries was sampled for aerobic and anaerobic culture (7–10 mL each) in BacT/Alert FA Plus and FN Plus resin bottles (bioMérieux Inc., Durham, NC, USA). Physicians selected the topical disinfectant such as ACHX, PVI, alcohol and others available in the ED, according to their personal preferences. A blood culture was considered contaminated if one or more of the following organisms were identified in one of two blood cultures: coagulase-negative *Staphylococci* (CoNS), *Propionibacterium acnes*, *Micrococci*, *Corynebacteria*, *Bacillus* species other than *Bacillus anthracis*, or *Clostridium perfringens*^10,15,16^. *Viridans streptococci* are regarded as contaminants based on the described criteria^10,15^, but they are not considered as contaminants at our institute because they were common causative agents of infective endocarditis. Polymicrobial cultures with a mixture of contaminant and true pathogens were regarded as contaminated^14^. A culture was defined as “negative” when bacterial growth was absent or when a bacterium was regarded by the attending microbiologist as having low pathogenicity.

### Statistical analysis

Categorical variables are described as frequencies and percentages (%) and continuous variables are shown as means with standard deviation (SD). Data were compared using one-way analyses of variance (ANOVA), χ^2^ and Fisher exact tests as appropriate. Differences in risk and robust 95% confidence intervals (CI) of contamination according to sites and topical disinfectants were estimated using univariate and multivariate analyses with modified least squares regression^17^. The same patients were considered as a random effect in the above model. Age, sex and disease status were adjusted as confounders in multivariate analyses. Because blood can be sampled from few sites, we also included in the category CV Other, blood sampled from recently inserted central venous (CV) catheter, venous and arterial catheters as well as implanted ports. Because we did not have many topical antiseptics to assess, we included only PVI or ACHX in analyses. We did not impute for missing values. Significance for all statistical findings was taken at *P* < 0.05. All data were statistically analyzed using SPSS version 25.0 software (IBM Corp., Armonk, NY, USA) or SAS software, version 9.4 (SAS Institute, Cary, NC, USA).

## Results

### Baseline characteristics

We analyzed 249 patients who were prospectively included in this study between August 1, 2018 and January 31, 2019. Thus, data from 249 patients and 483 pairs of blood cultures were analyzed. A total of 50 (20.1%) patients with potential contaminants were found in blood cultures, the most common of which was *Staphylococcus epidermidis* in 12 (24.0%), followed by *Staphylococcus hominis* in 4 (8.0%)*.* Two (0.8%) patients had a mixture of true bacteremia and contaminating isolates. Fifty-six (22.5%) patients had true bacteremia with *Escherichia coli* being the most prevalent microorganism in 17 (30.4%)*.* Cultured blood samples from 143 (57.4%) patients were identified as true negative. The most common source of infection in 22 (39.3%) patients with true bacteremia was the urinary tract, whereas pulmonary disease was the most prevalent in 17 (34.0%) and 52 (36.4%) patients with contaminated and true negative cultures, respectively. These two sources significantly differed among the three groups (pulmonary disease and urinary tract; both *P* = 0.001). Only one pair of blood samples was cultured from 15 patients. Table 1 shows other baseline characteristics of the three groups of patients.

**Table 1:** Characteristics of patients with blood cultures in emergency department.

Characteristics of patients	True bacteremia	Contamination	True negative	*P*
	n = 56	n = 50	n = 143	
Mean age, y (SD)	72 (11.2)	75.5 (10.2)	67.2 (18.3)	0.003
Male sex, n (%)	32 (57.1)	35 (70.0)	80 (55.9)	0.211
**Major comorbidities, n (%)**				
Malignancy	30 (53.6)	25 (50.0)	62 (43.4)	0.387
Diabetes mellitus	13 (23.2)	16 (32.0)	36 (25.2)	0.55
Hypertension	31 (55.4)	33 (66.0)	53 (37.1)	0.001
Previous stroke	4 (7.1)	3 (6.0)	18 (12.7)	0.296
Chronic renal insufficiency	6 (10.7)	4 (8.0)	10 (7.0)	0.688
Liver cirrhosis	1 (1.8)	3 (6.0)	1 (0.7)	0.071
Coronary artery diseases	9 (16.1)	4 (8.0)	13 (9.1)	0.29
Dementia	4 (7.3)	5 (10.0)	8 (5.6)	0.545
**Quick SOFA, n (%)**				
0	16 (28.6)	19 (38.0)	61 (42.7)	0.009
1	15 (26.8)	15 (30.0)	53 (37.1)	
2	19 (33.9)	11 (22.0)	27 (18.9)	
3	6 (10.7)	5 (10.0)	2 (1.4)	
**Origin of infection, n (%)**				
Central nervous system	2 (3.6)	2 (4.0)	5 (3.5)	0.987
Pulmonary	6 (10.7)	17 (34.0)	53 (37.1)	0.001
Cardiovascular system	3 (5.4)	0 0.0	7 (4.9)	0.27
Abdomen	11 (19.6)	14 (28.0)	32 (22.4)	0.581
Urinary tract	21 (37.5)	2 (4.0)	26 (18.2)	< 0.001
Skin	5 (8.9)	2 (4.0)	5 (3.5)	0.264
Other	8 (14.3)	13 (26.0)	15 (10.5)	0.027

*SD* standard deviation, *SOFA* sequential organ failure assessment, *ACHX* 1.0% alcohol/chlorhexidine gluconate, *CV* central venous; Femoral, femoral artery or vein; Other types, alcohol and benzalkonium; Other, recently inserted arterial catheter and implanted port; PVI, 10% aqueous povidone-iodine; Venous, venipuncture without catheter insertion; Venous catheter, recently inserted venous catheter.

Origin of infection means the cause of infection, as judged based on medical chart review including other cultures and various diagnostic modalities.

Central nervous system included meningitis, encephalitis, and brain abscess. Pulmonary included pneumonia, bronchitis, pleuritis, and upper respiratory infection. Cardiovascular system included endocarditis and pericarditis. Abdomen included cholangitis, gastroenteritis, cancer of gastrointestinal tract, hepatitis, cholecystitis, appendicitis, and pancreatitis. Urinary tract included pyelonephritis, cystitis, and prostatitis. Skin included decubitus, cellulitis, impetigo, and erysipelas. Other included febrile neutropenia and cases in which the source of infection could not be identified.

### Sites and topical antiseptics

Femoral arteries and veins tended to be sampled in most patients in all three groups, but at different ratios (Tables 1 and 2). Over 60% of blood samples from patients with contaminants was collected from femoral sites (mostly the femoral artery), whereas < 50% of blood samples from patients with negative cultures was collected from these sites. Blood sampled from a recently inserted central venous catheter conferred the greatest risk for contamination when taken as an independent factor. However, the number of samples was small (n = 15); thus, we classified this as “CV Other”.

**Table 2:** Blood culture sites and topical antiseptics.

	True bacteremia		Contamination		True negative	
	n = 111		n = 99		n = 273	
	First	Second	First	Second	First	Second
	n = 56	n = 55	n = 50	n = 49	n = 143	n = 130
**Site of blood sampling n (%)**						
CV other	9 (16.1)	10 (17.9)	8 (16.0)	6 (12.0)	27 (18.9)	29 (20.3)
Venous	12 (21.4)	11 (19.6)	12 (24.0)	9 (18.0)	56 (39.2)	43 (30.1)
Femoral	35 (62.5)	35 (62.5)	30 (60.0)	35 (70.0)	60 (42.0)	71 (49.7)
**Antiseptics, n (%)**						
PVI	36 (64.3)	35 (62.5)	47 (94.0)	47 (94.0)	102 (71.3)	89 (62.2)
ACHX	19 (33.9)	20 (35.7)	1 (2.0)	0	37 (25.9)	39 (27.3)
Other types	1 (1.8)	0	2 (4.0)	2 (4.0)	4 (2.8)	2 (1.4)
One pair	0	1 (1.8)	0	1 (2.0)	0	13 (9.1)

ACHX, 1.0% alcohol/chlorhexidine gluconate; CV Other, blood culture sample from recently inserted central venous catheter, venous and arterial catheters, implanted port; Femoral, femoral artery or vein; First, first pair of blood cultures; Other types, alcohol and benzalkonium; PVI, 10% aqueous povidone-iodine; Second, second two pairs of blood cultures; Venous, venipuncture without catheter insertion.

Topical antiseptics also significantly differed among the three groups (Table 2). The sites of > 90% of patients with contaminated blood cultures were disinfected with PVI, compared with < 75% of the patients in the other two groups. Other topical antiseptics comprising alcohol or benzalkonium were not included in the analysis because of small numbers (Table 2) and the possibility of confounding the results.

### Proportion of contamination by sites and topical antiseptics

With reference to ACHX, univariate analysis using modified least squares regression associated PVI with contamination (proportions of contamination associated with PVI and ACHX: 16.2% vs. 0.8%; risk difference, 15.4%; 95% CI 11.2–19.6; *P* < 0.001). With reference to blood collected from venous venipuncture sites, univariate analysis using modified least squares regression associated femoral sites or CV Other with contamination (Table 3).

**Table 3:** Univariate analysis using modified least squares regression.

	Contaminants (%)	Risk difference	95% CI	*P*
**Topical antiseptics**				
PVI	16.2	15.4	11.2–19.6	< 0.001
ACHX	0.8	-		
**Blood sampling sites**				
CV Other	21.2	19.0	4.9–33.2	0.009
Femoral	18.0	15.9	10.8–21.0	< 0.001
Venous	2.2	-		

ACHX, 1.0% alcohol/chlorhexidine gluconate; *CI* confidence interval; CV Other, blood culture sample from recently inserted central venous, venous and arterial catheters, implanted port; Femoral, femoral artery or vein; PVI, 10% aqueous povidone-iodine; Venous, venipuncture without catheter insertion.

Multivariate analysis showed that the proportion of contamination was higher for PVI than ACHX (adjusted risk difference, 12.9%; 95% CI 8.8–16.9; *P* < 0.001). Sites of blood sampling were also associated with contamination. The proportion of contamination was higher at femoral sites and CV Other, than at venous at sites (adjusted risk differences: 13.1% [95% CI 8.2–17.9; *P* < 0.001] and 17.3% [95% CI 3.6–31.0; *P* = 0.013], respectively. Sex was not significantly associated with contamination, however age was associated with contamination (Table 4).

**Table 4:** Multivariate analysis using modified least squares regression.

Parameter	Reference	Risk difference	95% CI	*P*
PVI	ACHX	12.9	8.8–16.9	< 0.001
CV Other	Venous	17.3	3.6–31.0	0.013
Femoral	Venous	13.1	8.2–17.9	< 0.001
Male	Female	2.3	-3.4–8	0.425
Age	per 10 years	1.8	0.3–3.2	0.015
**Antiseptics and blood sampling sites**				
PVI/CV Other	ACHX/Femoral	24.5	9.7–67.8	0.004
PVI/Femoral	ACHX/Femoral	20.3	13.5–27.0	< 0.001
PVI/Venous	ACHX/Femoral	2.2	−2.6 to 7.1	0.362
ACHX/CV Other	ACHX/Femoral	1.7	−2.6 to 6.0	0.444
ACHX/Venous	ACHX/Femoral	−1.1	−4.6 to 2.3	0.524
Male	Female	2.2	−3.5 to 7.9	0.447
Age	per 10 years	1.7	0.2–3.1	0.028

ACHX, 1.0% alcohol/chlorhexidine gluconate; *CI* confidence interval, CV Other, blood culture sample from recently inserted central venous, venous and arterial catheters, implanted port; Femoral, femoral artery or vein; PVI, 10% aqueous povidone-iodine; Venous, venipuncture without catheter insertion.

We also assessed associations between sites and topical antiseptics with contamination. With reference to femoral sites and ACHX, PVI and femoral sites, and PVI and CV Other were significantly associated with contamination, whereas PVI and venous, ACHX and CV Other and ACHX and venous sites were not (Table 4).

## Discussion

This single center, prospective observational study found that blood samples collected from femoral areas disinfected with PVI were significantly associated with contaminated blood cultures. The most common source of infection among patients with true bacteremia was the urinary tract, and most of such patients had pyelonephritis. In contrast, the most prevalent source of infection among patients with contaminated blood cultures was pulmonary disease, with most of such patients having aspiration pneumonia.

We also found that femoral puncture sites comprised an independent risk factor for blood culture contamination. Physicians tended to collect blood from femoral arteries or veins because it is easier than collecting from other sites. However femoral sites are colonized more often than other sites^18^ and these are associated with catheter-related bloodstream infection^19^. Internal jugular sites also confer risk for blood culture contamination^18,19^. One observational study in an intensive care setting identified higher contamination rates in cultures of blood sampled from recently inserted central lines compared with arterial lines and via direct peripheral venipuncture^20^. The contamination rate in the present study was the highest (53.3%) in 15 blood samples collected from central catheters that were recently inserted into the internal jugular vein. However, this number was too low to be statistically relevant.

Several reports have described associations between topical antiseptics and blood culture contamination^21,22^. A meta-analysis has found that blood culture contamination is more significantly reduced by ACHX than by PVI^7^. However physicians tended to disinfect puncture sites with PVI more frequently than ACHX at our hospital for the following reasons. Firstly, physicians and ED staff are more familiar with PVI than ACHX because it has been applied as a skin disinfectant for many years. Secondly, residents and medical students are not educated about blood culture procedures while at university.

Pneumonia was the most common illness among patients with contaminated and true negative blood cultures. Several studies of patients with community-acquired pneumonia (CAP) have found that blood cultures provide little diagnostic benefit^23–25^. One reason for the very high contamination rate at our hospital was due to mishandling of cultured blood samples from adult patients with CAP and isolated leukocytosis or fever. Blood samples should be cultured from selected immunocompromised patients, those with complicated urinary tract infection who are under antibiotic therapy at the time of blood collection, and patients with suspected endocarditis^24,26^.

Several strategies have been advocated to reduce rates of blood culture contamination. Sampling from various venipuncture sites, and reliance on a well-trained phlebotomy team can reduce these rates^6^. Furthermore, switching from PVI to ACHX or other topical antiseptics and informational intervention and feedback might reduce rates of blood culture contamination, even when physicians conduct phlebotomies^27,28^.

This study has several limitations. Our patient cohort was small and some parameters could not be conclusively determined. Nevertheless, specific injection sites and PVI were associated with significantly increased contamination rates. Some physicians were aware of this study proceeding within the ED and might have been more attentive when collecting blood than they might have been in wards. Physicians could select their preferred topical disinfectant for blood sampling, which might be a confounder because many studies have associated contamination more often with PVI than with ACHX. Blood collection sites prepared using the same disinfectant should be compared under the same conditions.

## Conclusions

This prospective observational study found that femoral puncture sites and PVI were independent risk factors for blood culture contamination. Other sites and antiseptics should be selected for skin disinfection before blood sampling to reduce culture contamination. Physicians should use ACHX when they collect blood culture sample from femoral puncture sites.

## Supplementary Information

Supplementary Information

## Data Availability

The datasets used and/or analyzed during the current study are available from the corresponding author on reasonable request.
