# Supplementary material for: Sites of blood collection and topical antiseptics associated with contaminated cultures: prospective observational study
Source: Sci Rep. 2021 Mar 18;11:6211. doi: 10.1038/s41598-021-85614-7 (PMC7973780; doi:10.1038/s41598-021-85614-7)
Supplement: Supplementary file 1 — Supplementary Information [file 41598_2021_85614_MOESM1_ESM.docx]

Supplementary table microorganisms isolated from blood cultures

| Date | **Site of blood sampling** | **Antiseptics** | Bacteria of frist set | **Site of blood sampling** | **Antiseptics** | Bacteria of second set | Decision | One pair and death |
| --- | --- | --- | --- | --- | --- | --- | --- | --- |
| 2018/8/1 | CV Other | PVI | Staphylococcus lugdunensis | Venous | PVI | negative | contamination | Death　13day |
| 2018/8/2 | CV Other | PVI | negative | Femoral | PVI | Staphylococcus epidermidis, Corynebacterium amycolatum | contamination | Death　2months |
| 2018/8/7 | CV Other | PVI | Bacillus cereus、Staphylococcus capitis | Femoral | PVI | Clostridium perfringens | contamination | Death　1month |
| 2018/8/7 | Femoral | PVI | Staphylococcus hominis ssp. hominis | Femoral | PVI | Staphylococcus hominis ssp. hominis | true positive |  |
| 2018/8/8 | Venous | PVI | negative | Femoral | PVI | Staphylococcus epidermidis | contamination |  |
| 2018/8/9 | Femoral | ACHX | Pseudomonas aeruginosa | Femoral | ACHX | Pseudomonas aeruginosa | true positive | Death　1day |
| 2018/8/9 | Venous | PVI | negative | Femoral | PVI | negative | true negative |  |
| 2018/8/9 | CV Other | PVI | negative |  |  |  | true negative | One pair |
| 2018/8/10 | Femoral | PVI | Staphylococcus epidermidis | Venous | PVI | negative | contamination |  |
| 2018/8/11 | Femoral | PVI | negative | Femoral | PVI | Staphylococcus caprae | contamination | Death　4month |
| 2018/8/12 | Venous | PVI | negative | Femoral | PVI | negative | true negative |  |
| 2018/8/12 | Femoral | PVI | negative | Femoral | PVI | negative | true negative |  |
| 2018/8/12 | Venous | PVI | negative |  |  |  | true negative | One pair |
| 2018/8/12 | Femoral | ACHX | negative | Venous | ACHX | negative | true negative |  |
| 2018/8/13 | Femoral | PVI | Escherichia coli | Femoral | PVI | *Escherichia coli、Staphylococcus hominis ssp. Hominis、Streptococcus sanguinis* | contamination |  |
| 2018/8/14 | Venous | Other types | negative | Femoral | Other types | negative | true negative | Death　7day |
| 2018/8/15 | Femoral | PVI | negative | Femoral | PVI | negative | true negative |  |
| 2018/8/15 | Femoral | PVI | *Staphylococcus hominis ssp. hominis* | Femoral | PVI | negative | contamination |  |
| 2018/8/15 | CV Other | PVI | negative | Venous | PVI | negative | true negative |  |
| 2018/8/16 | CV Other | PVI | negative | Venous | PVI | negative | true negative |  |
| 2018/8/17 | Venous | ACHX | *Streptococcus constellatus ssp. pharyngis* | Femoral | ACHX | *Streptococcus constellatus ssp. pharyngis* | true positive | Death　3month |
| 2018/8/17 | Femoral | PVI | negative |  |  |  | true negative | One pair |
| 2018/8/18 | CV Other | PVI | negative | Venous | PVI | *Streptococcus salivarius ssp. salivarius* | contamination |  |
| 2018/8/19 | Venous | PVI | *Streptococcus intermedius、Parvimonas（Micromonas） micros* | Femoral | PVI | *Parvimonas（Micromonas） micros* | true positive |  |
| 2018/8/20 | Femoral | PVI | negative |  |  |  | true negative | One pair 　Death2year |
| 2018/8/20 | Femoral | PVI | *Escherichia coli* | Femoral | PVI | *Escherichia coli* | true positive |  |
| 2018/8/24 | Femoral | PVI | negative | Femoral | PVI | *Staphylococcus hominis ssp. hominis* | contamination |  |
| 2018/8/25 | Femoral | PVI | *Escherichia coli* | CV Other | PVI | *Escherichia coli* | true positive |  |
| 2018/8/26 | Femoral | ACHX | *Escherichia coli* | Venous | ACHX | *Escherichia coli* | true positive |  |
| 2018/8/27 | Venous | PVI | negative | Femoral | PVI | *Staphylococcus epidermidis* | contamination | Death　6month |
| 2018/8/28 | Venous | PVI | negative |  |  |  | true negative | One pair |
| 2018/8/28 | Venous | PVI | *Streptococcus equisimilis* | Femoral | PVI | *Streptococcus equisimilis* | true positive |  |
| 2018/8/31 | Venous | PVI | negative |  |  |  | true negative | One pair |
| 2018/9/1 | Femoral | PVI | *Klebsiella oxytoca* | Femoral | PVI | *Klebsiella oxytoca* | true positive |  |
| 2018/9/1 | Venous | PVI | negative | Femoral | PVI | *Staphylococcus epidermidis* | contamination |  |
| 2018/9/2 | Femoral | ACHX | negative | Femoral | ACHX | negative | true negative |  |
| 2018/9/2 | CV Other | PVI | *Escherichia coli* | Femoral | PVI | *Escherichia coli* | true positive |  |
| 2018/9/3 | CV Other | PVI | negative | Femoral | PVI | negative | true negative |  |
| 2018/9/3 | CV Other | PVI | negative | Femoral | PVI | negative | true negative |  |
| 2018/9/4 | Venous | ACHX | negative | Venous | ACHX | negative | true negative |  |
| 2018/9/4 | Venous | ACHX | negative | Femoral | ACHX | negative | true negative |  |
| 2018/9/5 | Femoral | PVI | *Staphylococcus capitis* | Venous | PVI | negative | contamination |  |
| 2018/9/8 | Femoral | PVI | *Streptococcus gallolyticus spp. pasteurianus* | Femoral | PVI | *Streptococcus gallolyticus spp. pasteurianus* | true positive |  |
| 2018/9/8 | Venous | PVI | negative | Venous | PVI | negative | true negative |  |
| 2018/9/8 | Femoral | ACHX | negative | Femoral | ACHX | negative | true negative |  |
| 2018/9/8 | Femoral | PVI | *Staphylococcus epidermidis* | Femoral | PVI | negative | contamination | Death　1month |
| 2018/9/8 | Femoral | PVI | *Listeria monocytogenes* | Femoral | PVI | negative | true positive |  |
| 2018/9/10 | Venous |  | negative |  |  |  | true negative | One pair |
| 2018/9/11 | Venous | PVI | negative | Femoral | PVI | negative | true negative |  |
| 2018/9/12 | Venous | ACHX | negative | Venous | ACHX | negative | true negative |  |
| 2018/9/12 | Femoral | PVI | negative | Venous | PVI | negative | true negative |  |
| 2018/9/12 | CV Other | ACHX | *Bacteroides uniformis* | Femoral | ACHX | *Parabacteroides distasonis* | true positive |  |
| 2018/9/14 | Femoral | PVI | negative | Venous | PVI | negative | true negative |  |
| 2018/9/16 | Femoral | PVI | negative | Venous | PVI | negative | true negative | Death　2day |
| 2018/9/17 | Venous | PVI | *Klebsiella pneumoniae ssp. pneumoniae* | Femoral | PVI | *Staphylococcus capitis* | contamination |  |
| 2018/9/19 | CV Other | PVI | negative | CV Other | Other types | negative | true negative | Death　1month |
| 2018/9/20 | Venous | PVI | negative | Venous | PVI | negative | true negative |  |
| 2018/9/21 | Femoral | PVI | *Staphylococcus hominis ssp. hominis* | Femoral | ACHX | negative | contamination |  |
| 2018/9/21 | Femoral | PVI | negative | Femoral | PVI | negative | true negative | Death　1month |
| 2018/9/22 | Femoral | PVI | *Staphylococcus epidermidis* | Venous | PVI | negative | contamination |  |
| 2018/9/22 | Venous | PVI | negative | Venous | PVI | negative | true negative |  |
| 2018/9/22 | Femoral | ACHX | *Staphylococcus epidermidis, Propionibacterium acnes* | CV Other | PVI | *Staphylococcus epidermidis* | contamination |  |
| 2018/9/23 | Venous | PVI | negative | Femoral | PVI | *Staphylococcus hominis ssp. hominis* | contamination | Death　2year |
| 2018/9/23 | CV Other | PVI | negative | Femoral | PVI | negative | true negative |  |
| 2018/9/24 | Femoral | PVI | negative | Femoral | PVI | *Staphylococcus hominis ssp. Hominis, Staphylococcus epidermidis* | contamination |  |
| 2018/9/25 | Femoral | PVI | *Staphylococcus epidermidis* | Femoral | PVI | *Staphylococcus lugdunensis* | contamination |  |
| 2018/9/26 | Femoral | ACHX | negative | Venous | ACHX | negative | true negative |  |
| 2018/9/26 | Femoral | ACHX | negative | Femoral | ACHX | negative | true negative | Death　1month |
| 2018/9/28 | Venous | PVI | negative | Femoral | PVI | negative | true negative |  |
| 2018/9/29 | Femoral | PVI | negative | Femoral | PVI | negative | true negative |  |
| 2018/9/30 | Femoral | PVI | negative |  |  |  | true negative | One pair |
| 2018/10/1 | Venous | PVI | negative | Venous | PVI | negative | true negative |  |
| 2018/10/1 | Femoral | PVI | negative | Femoral | PVI | negative | true negative |  |
| 2018/10/1 | Femoral | PVI | negative | Venous | PVI | negative | true negative |  |
| 2018/10/1 | Venous | PVI | *Morganella morganii ssp. morganii* | Venous | PVI | *Staphylococcus hominis ssp. hominis* | contamination |  |
| 2018/10/4 | Venous | PVI | negative | CV Other | PVI | negative | true negative |  |
| 2018/10/4 | Femoral | PVI | negative | Femoral | PVI | *Staphylococcus warneri* | contamination | Death　60day |
| 2018/10/5 | Venous | PVI | negative | Femoral | PVI | negative | true negative |  |
| 2018/10/5 | Femoral | PVI | negative |  |  |  | true negative | One pair |
| 2018/10/6 | Femoral | PVI | *Veillonella species* | Femoral | PVI | *Streptococcus intermedius* | true positive |  |
| 2018/10/6 | Venous | PVI | negative | Femoral | PVI | negative | true negative |  |
| 2018/10/7 | Femoral | ACHX | *Escherichia coli* | Venous | ACHX | *Escherichia coli* | true positive |  |
| 2018/10/7 | Femoral | ACHX | negative | Femoral | ACHX | negative | true negative |  |
| 2018/10/8 | Venous | PVI | negative | Femoral | PVI | negative | true negative |  |
| 2018/10/9 | Venous | PVI | *Enterococcus faecalis* | Venous | PVI | *Enterococcus faecalis* | true positive | Death　2year |
| 2018/10/10 | Femoral | PVI | negative | Femoral | PVI | negative | true negative |  |
| 2018/10/10 | Femoral | PVI | *Clostridium perfringens* | Venous | PVI | *Clostridium perfringens* | true positive | Death　1day |
| 2018/10/12 | Femoral | ACHX | negative | Venous | ACHX | negative | true negative |  |
| 2018/10/12 | Femoral | ACHX | *Pseudomonas aeruginosa、Enterococcus faecalis* | Venous | ACHX | *Pseudomonas aeruginosa* | true positive |  |
| 2018/10/12 | Venous | PVI | negative | Venous | PVI | negative | true negative |  |
| 2018/10/14 | Femoral | PVI | *Streptococcus pyogenes(groupA)* | Femoral | PVI | *Streptococcus pyogenes(groupA)* | true positive |  |
| 2018/10/14 | Femoral | PVI | *Bacteroides thetaiotaomicron、Clostridium innocuum* | Femoral | PVI | *Bacteroides thetaiotaomicron* | contamination |  |
| 2018/10/17 | Femoral | PVI | *Staphylococcus hominis ssp. hominis* | Venous | PVI | negative | contamination |  |
| 2018/10/19 | Femoral | PVI | negative | Venous | PVI | negative | true negative |  |
| 2018/10/19 | Femoral | PVI | negative | Venous | PVI | negative | true negative |  |
| 2018/10/20 | Femoral | PVI | negative | Femoral | PVI | negative | true negative |  |
| 2018/10/21 | Femoral | PVI | *Staphylococcus hominis ssp. hominis* | Venous | PVI | negative | contamination |  |
| 2018/10/21 | Femoral | PVI | *Bacillus cereus* | Venous | PVI | negative | contamination |  |
| 2018/10/22 | Femoral | ACHX | negative | Venous | ACHX | negative | true negative |  |
| 2018/10/22 | Venous | PVI | negative | Femoral | PVI | negative | true negative |  |
| 2018/10/22 | Venous | PVI | negative | Venous | PVI | negative | true negative |  |
| 2018/10/24 | Femoral | PVI | negative | CV Other | PVI | negative | true negative |  |
| 2018/10/24 | Femoral | ACHX | negative | Venous | PVI | negative | true negative |  |
| 2018/10/25 | Femoral | PVI | negative | Femoral | PVI | negative | true negative |  |
| 2018/10/25 | Femoral | ACHX | Escherichia coli (ESBL)、Streptococcus dysgalactiae ssp. Equisimilis、Streptococcus agalactiae(groupB) | Femoral | ACHX | *Escherichia coli (ESBL)、Pseudomonas aeruginosa、Streptococcus dysgalactiae ssp. Equisimilis、Streptococcus agalactiae(groupB)* | true positive | Death　1year |
| 2018/10/26 | Femoral | PVI | negative | Venous | ACHX | negative | true negative |  |
| 2018/10/29 | Venous | PVI | *Escherichia coli,Streptococcus species* | Femoral | PVI | *Escherichia coli,Streptococcus species* | true positive |  |
| 2018/10/31 | Venous | ACHX | negative | Femoral | ACHX | negative | true negative |  |
| 2018/10/31 | Femoral | PVI | *Escherichia coli* | Femoral | PVI | *Escherichia coli* | true positive |  |
| 2018/11/1 | Venous | PVI | negative | Femoral | PVI | negative | true negative |  |
| 2018/11/1 | Venous | ACHX | *Escherichia coli* | Venous | ACHX | *Escherichia coli* | true positive |  |
| 2018/11/1 | Venous | PVI | negative | Venous | PVI | negative | true negative |  |
| 2018/11/2 | Venous | ACHX | negative | Femoral | ACHX | negative | true negative |  |
| 2018/11/3 | Femoral | PVI | negative | Venous | PVI | negative | true negative |  |
| 2018/11/4 | Femoral | ACHX | negative | Venous | ACHX | negative | true negative |  |
| 2018/11/5 | Venous | PVI | negative | Femoral | PVI | negative | true negative |  |
| 2018/11/5 | Femoral | PVI | *Escherichia coli* | Femoral | PVI | *Escherichia coli* | true positive | Death　3month |
| 2018/11/6 | Venous | ACHX | negative | Venous | ACHX | negative | true negative |  |
| 2018/11/7 | Venous | PVI | negative | Venous | PVI | negative | true negative |  |
| 2018/11/7 | Venous | PVI | negative | Femoral | PVI | negative | true negative |  |
| 2018/11/8 | Venous | ACHX | negative | Venous | ACHX | negative | true negative |  |
| 2018/11/8 | CV Other | PVI | negative | Venous | PVI | negative | true negative |  |
| 2018/11/9 | Femoral | Other types | negative | Venous | ACHX | negative | true negative |  |
| 2018/11/9 | Venous | ACHX | negative | Femoral | ACHX | negative | true negative |  |
| 2018/11/10 | Venous | PVI | negative | Venous | PVI | negative | true negative |  |
| 2018/11/11 | Venous | PVI | *Enterobacter cloacae complex* | Venous | PVI | *Enterobacter cloacae complex* | true positive | Death　6month |
| 2018/11/13 | Femoral | PVI | negative | Femoral | PVI | negative | true negative |  |
| 2018/11/13 | Venous | PVI | *Escherichia coli* | Femoral | PVI | negative | true positive |  |
| 2018/11/13 | Femoral | PVI | negative | Venous | PVI | negative | true negative |  |
| 2018/11/13 | Venous | PVI | negative | Venous | PVI | negative | true negative |  |
| 2018/11/14 | Femoral | PVI | *Streptococcus agalactiae(groupB)* | Venous | PVI | negative | true positive | Death　2year |
| 2018/11/14 | Femoral | ACHX | *Escherichia coli* | CV Other | PVI | *Escherichia coli* | true positive |  |
| 2018/11/16 | Venous | ACHX | *Serratia marcescens* | CV Other | ACHX | *Serratia marcescens* | true positive |  |
| 2018/11/16 | Femoral | PVI | negative | Femoral | PVI | negative | true negative |  |
| 2018/11/17 | Femoral | PVI | *Bacillus cereus、Staphylococcus epidermidis* | Femoral | PVI | *Enterococcus faecalis、Staphylococcus epidermidis* | contamination |  |
| 2018/11/17 | Venous | PVI | negative | Femoral | PVI | negative | true negative |  |
| 2018/11/18 | Femoral | ACHX | negative | Venous | ACHX | negative | true negative |  |
| 2018/11/19 | Venous | ACHX | negative | Venous | ACHX | negative | true negative |  |
| 2018/11/19 | Femoral | PVI | *Staphylococcus epidermidis* | Femoral | PVI | *Micrococcus luteus* | contamination |  |
| 2018/11/20 | Venous | PVI | negative | Femoral | PVI | negative | true negative |  |
| 2018/11/20 | Venous | ACHX | *Streptococcus pneumoniae (PSSP)* | Femoral | ACHX | *Streptococcus pneumoniae (PSSP)* | true positive |  |
| 2018/11/20 | Femoral | PVI | negative | Femoral | ACHX | negative | true negative |  |
| 2018/11/23 | Femoral | PVI | *Enterococcus faecium* | Femoral | PVI | *Staphylococcus caprae* | contamination |  |
| 2018/11/25 | Venous | PVI | negative | Venous | PVI | negative | true negative |  |
| 2018/11/26 | Femoral | PVI | *Gram-negative bacilli* |  |  |  | true positive | One pair Death　2day |
| 2018/11/26 | Venous | PVI | negative | Femoral | PVI | negative | true negative |  |
| 2018/11/26 | Venous | PVI | negative | Femoral | PVI | negative | true negative |  |
| 2018/11/27 | Venous | PVI | negative | Femoral | PVI | negative | true negative |  |
| 2018/11/28 | Venous | PVI | negative | Femoral | PVI | negative | true negative |  |
| 2018/11/28 | Venous | ACHX | *Klebsiella pneumoniae ssp. pneumoniae* | CV Other | ACHX | *Klebsiella pneumoniae ssp. pneumoniae* | true positive | Death　7day |
| 2018/11/29 | Venous | PVI | negative | Femoral | PVI | negative | true negative |  |
| 2018/11/30 | Venous | PVI | negative | Femoral | ACHX | negative | true negative |  |
| 2018/11/30 | Venous | ACHX | *Escherichia coli* | CV Other | ACHX | *Escherichia coli* | true positive |  |
| 2018/12/1 | Venous | PVI | negative | Venous | PVI | negative | true negative |  |
| 2018/12/1 | Femoral | PVI | negative | Femoral | PVI | negative | true negative |  |
| 2018/12/1 | Venous | ACHX | negative | Venous | ACHX | negative | true negative |  |
| 2018/12/1 | Venous | PVI | *Klebsiella pneumoniae ssp. pneumoniae* | Femoral | PVI | Klebsiella pneumoniae ssp. Pneumoniae、Staphylococcus hominis ssp. hominis | true positive |  |
| 2018/12/1 | CV Other | PVI | negative | CV Other | PVI | negative | true negative |  |
| 2018/12/4 | Femoral | PVI | negative | Venous | PVI | negative | true negative |  |
| 2018/12/5 | Femoral | ACHX | negative | Femoral | ACHX | negative | true negative |  |
| 2018/12/5 | Venous | ACHX | negative | Femoral | ACHX | negative | true negative |  |
| 2018/12/5 | Venous | ACHX | negative | Femoral | ACHX | negative | true negative |  |
| 2018/12/7 | Femoral | PVI | *Morganella morganii ssp. Morganii、Streptococcus anginosus* | Femoral | PVI | Morganella morganii ssp. Morganii、Streptococcus anginosus | true positive | Death　7day |
| 2018/12/8 | Femoral | PVI | negative | Venous | PVI | negative | true negative |  |
| 2018/12/8 | Femoral | ACHX | *Streptococcus intermedius* | Venous | ACHX | *Streptococcus intermedius* | true positive |  |
| 2018/12/8 | Femoral | PVI | *Streptococcus equisimilis、Staphylococcus aureus (MRSA)* | Femoral | PVI | Streptococcus equisimilis、Staphylococcus aureus (MRSA) | true positive |  |
| 2018/12/9 | Femoral | PVI | negative | Femoral | PVI | negative | true negative |  |
| 2018/12/12 | Venous | PVI | negative | Venous | PVI | negative | true negative |  |
| 2018/12/14 | Femoral | PVI | negative | Femoral | PVI | negative | true negative |  |
| 2018/12/16 | Venous | ACHX | negative | Venous | ACHX | negative | true negative | Death　7day |
| 2018/12/19 | Femoral | PVI | negative | Femoral | PVI | negative | true negative |  |
| 2018/12/20 | Femoral | PVI | negative | Femoral | PVI | negative | true negative |  |
| 2018/12/20 | Femoral | PVI | *Staphylococcus epidermidis* | Venous | PVI | negative | contamination | Death　39day |
| 2018/12/20 | Venous | PVI | negative | Femoral | PVI | *Staphylococcus epidermidis* | contamination |  |
| 2018/12/21 | Femoral | PVI | negative | Femoral | PVI | negative | true negative |  |
| 2018/12/21 | Femoral | PVI | *Escherichia coli* | Femoral | PVI | *Escherichia coli* | true positive |  |
| 2018/12/22 | Femoral | PVI | negative |  |  |  | true negative | One pair |
| 2018/12/22 | Femoral | PVI | negative | Venous | PVI | negative | true negative |  |
| 2018/12/24 | Femoral | ACHX | *Staphylococcus aureus (MRSA)* | Venous | ACHX | *Staphylococcus aureus (MRSA)* | true positive |  |
| 2018/12/24 | Femoral | PVI | *Escherichia coli* | Venous | PVI | *Escherichia coli* | true positive |  |
| 2018/12/24 | Venous | PVI | negative |  |  |  | true negative | One pair |
| 2018/12/24 | Femoral | PVI | *Staphylococcus simulans* | Femoral | PVI | *Staphylococcus simulans* | contamination |  |
| 2018/12/24 | Venous | PVI | negative | CV Other | PVI | negative | true negative |  |
| 2018/12/25 | Femoral | ACHX | negative | CV Other | PVI | negative | true negative |  |
| 2018/12/25 | Venous | PVI | negative | Femoral | PVI | *Staphylococcus epidermidis、Glucose nonfermentative GNR* | contamination |  |
| 2018/12/29 | Venous | ACHX | negative | Femoral | PVI | *Staphylococcus caprae* | contamination |  |
| 2018/12/29 | Venous | ACHX | negative | Femoral | ACHX | negative | true negative |  |
| 2018/12/29 | Venous | PVI | negative | Femoral | PVI | negative | true negative |  |
| 2018/12/29 | Femoral | PVI | *Staphylococcus aureus (MSSA)* | Femoral | PVI | *Staphylococcus aureus (MSSA)* | true positive | Death　2year |
| 2018/12/30 | Venous | PVI | negative | Femoral | PVI | negative | true negative |  |
| 2018/12/31 | Femoral | PVI | *Staphylococcus caprae* | CV Other | PVI | negative | contamination |  |
| 2018/12/31 | Femoral | Other types | negative | Femoral | Other types | negative | true negative |  |
| 2018/12/31 | Femoral | ACHX | negative | Femoral | ACHX | negative | true negative |  |
| 2018/12/31 | Femoral | PVI | *Proteus mirabilis* | Femoral | PVI | *Proteus mirabilis* | true positive | Death　2day |
| 2019/1/1 | CV Other | Other types | *Staphylococcus aureus (MSSA)* | Venous | ACHX | *Staphylococcus aureus (MSSA)* | true positive | Death　4month |
| 2019/1/1 | Venous | ACHX | negative | Femoral | ACHX | negative | true negative |  |
| 2019/1/2 | Femoral | PVI | *Enterobacter cloacae complex* | Femoral | PVI | *Enterobacter cloacae complex* | true positive | Death　1month |
| 2019/1/2 | Femoral | PVI | *Gram-negative bacilli* | Femoral | PVI | *Gram-negative bacilli* | true positive | Unknown |
| 2019/1/2 | Femoral | PVI | negative | Venous | PVI | negative | true negative |  |
| 2019/1/2 | Femoral | PVI | negative | Venous | PVI | negative | true negative |  |
| 2019/1/3 | Femoral | PVI | Klebsiella oxytoca、Enterococcus faecalis | Femoral | PVI | Klebsiella oxytoca、Enterococcus faecalis | true positive | Death　5month |
| 2019/1/3 | Femoral | PVI | *Klebsiella pneumoniae ssp. pneumoniae* | Venous | PVI | *Klebsiella pneumoniae ssp. pneumoniae* | true positive |  |
| 2019/1/5 | Femoral | PVI | negative | Femoral | PVI | *Staphylococcus epidermidis* | contamination |  |
| 2019/1/5 | Venous | PVI | *Klebsiella oxytoca(ESBL)、Enterococcus faecalis、Morganella morganii ssp. morganii* | Femoral | PVI | Klebsiella oxytoca(ESBL)、Enterococcus faecalis、Morganella morganii ssp. morganii | true positive |  |
| 2019/1/5 | Venous | PVI | negative | Femoral | PVI | *Staphylococcus hominis ssp. Hominis、Staphylococcus epidermidis* | contamination |  |
| 2019/1/6 | Venous | PVI | negative |  |  |  | true negative | One pair |
| 2019/1/7 | Femoral | PVI | negative | Femoral | PVI | negative | true negative |  |
| 2019/1/8 | Femoral | PVI | *Escherichia coli* | Femoral | PVI | *Escherichia coli* | true positive | Death　2year |
| 2019/1/9 | Femoral | PVI | *Staphylococcus epidermidis* | CV Other | PVI | *Propionibacterium acnes* | contamination |  |
| 2019/1/9 | Femoral | PVI | negative | Femoral | PVI | *Staphylococcus epidermidis* | contamination |  |
| 2019/1/9 | Venous | PVI | negative | Femoral | PVI | negative | true negative |  |
| 2019/1/10 | Femoral | ACHX | negative | Femoral | ACHX | negative | true negative |  |
| 2019/1/10 | Venous | ACHX | negative | Femoral | ACHX | *Raoultella planticola* | true positive |  |
| 2019/1/11 | Femoral | ACHX | *Enterobacter cloacae complex* | Femoral | ACHX | Enterobacter cloacae complex,Enterococcus faecalis,Staphylococcus aureus (MSSA) | true positive | Death　40day |
| 2019/1/11 | Venous | ACHX | negative | Femoral | ACHX | negative | true negative |  |
| 2019/1/12 | Venous | ACHX | negative | Femoral | ACHX | negative | true negative |  |
| 2019/1/12 | Venous | ACHX | *Staphylococcus aureus (MRSA)* | Venous | ACHX | *Staphylococcus aureus (MRSA)* | true positive |  |
| 2019/1/13 | Femoral | PVI | negative | Femoral | PVI | *Staphylococcus capitis* | contamination |  |
| 2019/1/13 | Venous | PVI | negative | Femoral | PVI | negative | true negative |  |
| 2019/1/13 | Femoral | PVI | *Staphylococcus epidermidis* | Femoral | PVI | negative | contamination |  |
| 2019/1/14 | Venous | ACHX | *Enterococcus faecalis* | Venous | ACHX | *Enterococcus faecalis* | true positive |  |
| 2019/1/14 | Venous | PVI | negative | Venous | PVI | negative | true negative |  |
| 2019/1/14 | Venous | Other types | negative |  |  |  | true negative | One pair |
| 2019/1/14 | Venous | PVI | *Staphylococcus epidermidis* | Femoral | PVI | negative | contamination |  |
| 2019/1/15 | Venous | PVI | negative | Femoral | PVI | negative | true negative |  |
| 2019/1/15 | CV Other | PVI | *Staphylococcus auricularis* | Femoral | PVI | negative | contamination | Death　2year |
| 2019/1/15 | Venous | ACHX | negative | Venous | ACHX | negative | true negative |  |
| 2019/1/16 | Femoral | PVI | negative | Femoral | PVI | *Staphylococcus capitis* | contamination |  |
| 2019/1/16 | Venous | PVI | negative | Femoral | PVI | negative | true negative |  |
| 2019/1/17 | Femoral | PVI | negative | Venous | PVI | negative | true negative |  |
| 2019/1/18 | Femoral | ACHX | negative | Femoral | PVI | *Staphylococcus epidermidis* | contamination | Death　27day |
| 2019/1/18 | CV Other | PVI | *Staphylococcus aureus (MSSA)* | Femoral | ACHX | *Staphylococcus aureus (MSSA)* | true positive |  |
| 2019/1/19 | Venous | PVI | negative | Femoral | PVI | negative | true negative |  |
| 2019/1/19 | Venous | PVI | negative | Venous | PVI | negative | true negative |  |
| 2019/1/19 | Venous | ACHX | negative | Femoral | ACHX | negative | true negative |  |
| 2019/1/21 | CV Other | PVI | *Staphylococcus epidermidis* |  |  |  | contamination | One pair Death　1day |
| 2019/1/22 | CV Other | PVI | negative | Femoral | PVI | negative | true negative |  |
| 2019/1/24 | Femoral | PVI | negative | Femoral | PVI | negative | true negative |  |
| 2019/1/24 | Venous | PVI | negative | Femoral | PVI | negative | true negative |  |
| 2019/1/24 | Femoral | ACHX | negative | Femoral | ACHX | negative | true negative |  |
| 2019/1/24 | Femoral | PVI | negative | Venous | PVI | negative | true negative |  |
| 2019/1/25 | CV Other | PVI | *Staphylococcus capitis* | Femoral | PVI | *Staphylococcus epidermidis* | contamination |  |
| 2019/1/28 | Venous | ACHX | negative | Venous | ACHX | negative | true negative |  |
| 2019/1/28 | Femoral | PVI | *Escherichia coli (ESBL)* | Femoral | PVI | *Escherichia coli (ESBL)* | true positive |  |
| 2019/1/28 | Femoral | ACHX | negative | Venous | ACHX | negative | true negative |  |
| 2019/1/29 | Venous | PVI | *Staphylococcus capitis* | Venous | PVI | negative | contamination |  |
| 2019/1/29 | Femoral | PVI | negative | Femoral | PVI | *Bacillus species、Staphylococcus epidermidis* | contamination |  |
| 2019/1/31 | Venous | PVI | negative | Venous | PVI | negative | true negative |  |
| 2019/1/31 | Venous | PVI | negative | Femoral | PVI | *Staphylococcus epidermidis* | contamination |  |
